# State-of-the-art of invasive Group A Streptococcus infection in children: protocol for a scoping review of the literature with a focus on predictors of invasive infection

**DOI:** 10.12688/f1000research.130989.1

**Published:** 2023-02-24

**Authors:** Francesco Mariani, Laura Martino, Carolina Gentili, Valentina Pulcinelli, Piero Valentini, Danilo Buonsenso

**Affiliations:** 1 Department of Woman and Child Health and Public Health, Fondazione Policlinico Universitario A. Gemelli IRCCS, Rome, Italy, Roma, Italy

**Keywords:** streptococcus; group A streptococcus; iGAS

## Abstract

**Background:** Invasive group A streptococcus infection (iGAS) is a serious, sometimes life-threatening condition, with high case fatality rates and high morbidity whose incidence is greatly increased in the last years. Despite the increasing importance and frequency of this condition, at the best of our knowledge, no previous reviews have been published focusing on the risk factors for the development of this condition and its early clinical features. This paper reports the study protocol for a scoping review that aims to analyze the early signs and clinical features of invasive group A streptococcus disease in children, to recognize the prodromal stage of the disease.

**Methods:** Comprehensive research combining the terms pediatric and invasive group A streptococcus infection has been performed on PubMed and SCOPUS to identify potential eligible studies. The search strategy for PubMed will be available in this paper. Two reviewers will screen first the abstract and subsequently the full text to identify eligible articles according to the predefined inclusion criteria. Divergences between the reviewers will be resolved by discussion (with a third author if necessary). Two review authors will extract data independently, everyone on a different Excel spreadsheet. Each researcher will be blinded to the decision of the other researcher. When the process will be completed, in case of discordance, any disagreement will be identified and resolved through discussion (with a third author if necessary).

**Dissemination:** The findings of this review will be published in a peer-reviewed journal.

## Introduction

Streptococcus Pyogenes (Group A Streptococcus, GAS) can cause different infections, ranging from minor illnesses such as pharyngitis and superficial skin infections to severe diseases. An invasive disease is defined as the isolation of GAS from a normally sterile site of the body and it occurs when bacteria spreads throughout the bloodstream, the cerebrospinal fluid, the lungs and soft tissue. Group A Streptococcus Invasive infection (iGAS) is a serious, sometimes life-threatening condition, with high case fatality rates and high morbidity.

According to CDC Active Bacterial Core surveillance reports, overall invasive GAS incidence increased every year from 2012 to 2019; in 2020, especially during the first months of the pandemic period, the incidence of invasive disease saw an historical drop and the change was greatest for children aged 5 to 17 years. Unexpectedly, the preliminary data from the surveillance reports of 2022 showed a monthly increase of the incidence of iGAS infection in children from September to November, thus leading the CDC to issue a health advisory. Different European countries reported to ECDC an increase in iGAS disease in children aged less than 10 years from September 2022, with Ireland, France and UK reporting several deaths, too. It’s likely that this increase in incidence of iGAS is related to the high circulation of respiratory viruses, especially Respiratory Syncytial Virus (RSV) and seasonal influenza, since the coinfection of viruses can lead more easily to invasive disease.

The most common clinical presentations of iGAS during the pediatric age include bacteremia, soft tissue infections, Streptococcal toxic shock syndrome (STSS) and necrotizing fasciitis. ^1^ The presence of one other child living in the same house, a varicella zoster virus infection and the use of nonsteroidal anti-inflammatory drugs have been reported in literature as risk factors for iGAS. ^2^^,^^3^ Due to the extreme variety of severity of GAS infection, the early diagnosis of invasive disease is often challenging.

This scoping review aims to analyze the early signs and clinical features of invasive group A streptococcus disease in children, to recognize the prodromal stage of the disease and quickly start the appropriate antibiotic therapy and supportive care.

## Review questions

The main review question will be “which are the predictors of clinical iGAS”?

This review will also assess the following sub-questions:

1. What is known about the epidemiology of iGAS?
2. Which are the most frequently reported clinical characteristics of different iGAS?
3. Which outcomes are reported in literature about the different types of iGAS (pneumonia, meningitis, sepsis and abscesses)?

## Inclusion criteria

### Participants

This review will include studies performed on children and adolescents (younger than 18 years) with a confirmed diagnosis of iGAS defined as a laboratory isolation of GAS from any normal sterile site or isolation of GAS from a non-sterile site in patients with necrotizing fasciitis or streptococcal toxic shock syndrome. ^4^ We will include children diagnosed with pneumonia, sepsis, abscesses or meningitis, due to GAS invasion.

### Concept

The main concept of this review will be the iGAS in all its aspects.

### Context

Considering the severity of the disease, we will not expect to find articles involving patients not hospitalized so we will include only inpatients.

### Type of sources

This review will include both randomized controlled trials and non-randomized controlled trials. All the types of observational studies, prospective and retrospective (including case-control, cohort and cross-sectional studies, small case series or single case reports) will be included.

## Methods

### Search strategy

The search will be performed by one reviewer. We started our research in January 2023 in the following bibliographic databases: PubMed and SCOPUS. There will be no date restrictions. Only articles written in English will be included. The search strategy will include the following word: “pediatric”, “iGAS (and its possible clinical manifestations)” and “group A Streptococcus Pyogenes”. Patients younger than 18 years of age will be considered as children or pediatric patients. The search strategy for PubMed is available in the extended data section of this protocol ^5^; the terms used for this search were adapted for use with other bibliographic database.

If the final analysis were to be performed six months after the bibliographic search, the search string will be launched again to evaluate the presence of new studies to be included in the work.

### Study selection

After the search, the studies will be exported to Rayyan. A first screen to exclude duplicates will be performed by one author.

Titles and/or abstracts of studies retrieved using the search strategy will be screened independently by two reviewers to identify studies that could be inserted in the review. Full texts of potentially eligible studies will be retrieved and independently assessed for eligibility by two reviewers. Each researcher will be blinded to the decision of the other researcher. Any disagreement between them over the eligibility of studies will be resolved through discussion and, in case of further disagreement, by discussion with a third reviewer.

All the studies that will not meet the inclusion criteria will be excluded and a table with the reason why those studies were excluded will be inserted in the final manuscript.

The results of the search will be reported in the PRISMA flow diagram.

### Data extraction

Two review authors will extract data independently, everyone on a different Excel spreadsheet. Each researcher will be blinded to the decision of the other researcher. When the process will be completed, in case of discordance, any disagreement will be identified and resolved through discussion (with a third author if necessary).

An Excel file will be used to store data. When available, extracted information will include:

1. study general features: title, author, year of publication, type of study, number of patients included in the study, geographical area where the study has been performed
2. participant general features: sample size of each group, nationality, age, socio-economic status, comorbidities
3. previous clinical manifestations (during the 30 days preceding the diagnosis of iGAS): fever (including days), sore throat, vomiting, rashes, cough and others, known pharyngeal swab positive for Group A streptococcus, and others
4. clinical manifestation of the condition: fever (including days), neurological signs, vomiting, rashes, cough and others
5. a concomitant or previous (during the 30 days preceding the diagnosis of iGAS) viral infections microbiologically confirmed
6. main imaging findings: type of lung involvement at chest X-Ray and/or CT scan, type of CNS involvement at CT scan or MRI, type of skin involvement evaluated by ultrasound or CT scan or MRI, heart (US or CT or MRI)
7. GAS localizations (e.g., lung, central nervous system, blood or skin)
8. characteristics of eventual antimicrobial treatments performed during the 30 days preceding the diagnosis of iGAS (length of therapy, when this has been started and which antibiotic was used)
9. characteristics of eventual antimicrobial treatments performed during the iGAS (length of therapy, when this has been started and which antibiotic was used)
10. adjunctive treatments performed and length of therapy during the iGAS (e.g., steroids or other immunomodulatory medications)
11. outcomes (death, survival; survival with or without sequelae; type of sequelae)

### Data analysis and presentation

To report our findings, we will follow Preferred Reporting Items for Systematic reviews and Meta-Analyses extension for Scoping Reviews (PRISMA-ScR) Checklist.

We will produce a narrative synthesis of the findings from the studies included in the review describing the results we have obtained and providing our opinion on their interpretation. The selection of the studies for the main narrative synthesis will be performed preferring articles in which the clinical history before the iGAS has been reported; for this part we will include only articles in which the risk factors of iGAS development was evaluated trough multivariable analysis. This selection is motived by the fact that our first interest is the identification of iGAS predictors. If after this selection will be included more than 100 records, original article and those published in the last 5 years will be preferred.

We will also use tables and charts to summarize both study characteristics and the most important clinical, diagnostics, treatments and outcomes data.

More specifically, we will summarize our findings using different tables. The first one will include the characteristics of included studies (number of studies, study design, year of publication, characteristics of the study populations, and countries where studies were conducted) and the participant general features. Then we will provide different tables or figures summarizing main data about clinical presentation, imaging characteristics, GAS localization, treatments performed, outcomes and predictors of iGAS.

This way we hope we will be able to provide a useful document containing what is currently known of pediatric iGAS with the aim of informing clinicians about the general characteristics of these conditions, focusing on risk factors and early clinical features, and guide future research projects to fill current gaps.

## Study status

We launched our research and performed the abstract screening. We are going to start the full text screening.

## Patient and public involvement

There was no direct patient and public involvement in this review. However, the key questions that led us implementing this research project were inspired by public discussions started by family associations in the media, highlighting the importance of better comprehension of how iGAS can be recognized earlier in the disease course (before clinical conditions deteriorates and cannot be controlled anymore), or iGAS may also be prevented if this complication is a consequence of a previous unrecognized and untreated GAS infection.

## Strengths and limitations of this study

• A scoping review can represent the best way to report on the types of evidence that are published in a certain field and our paper will provide an overview of iGAS, focusing on predictors of invasive infection in children.
• A scoping review can represent the best way to examine this field to guide future research on this topic.
• To report our findings, we will follow the Preferred Reporting Items for Systematic reviews and Meta-Analyses extension for Scoping Reviews (PRISMA-ScR) Checklist to ensure methodological strength to our paper.
• Only two databases were screened, and only English paper will be considered limiting the number of papers that will be included.
• No critical appraisal neither risk of bias of the included studies will be performed, considering the exploratory role of this paper.

## Data Availability

No data are associated with this article. Figshare: Supplementary Material.docx, https://doi.org/10.6084/m9.figshare.22094219.v1. ^5^ This project contains the following extended data:
- Supplementary Material.docx (Search strategy for PubMed) Data are available under the terms of the Creative Commons Zero “No rights reserved” data waiver (CC0 1.0 Public domain dedication).
