# Supplementary material for: State-of-the-art of invasive Group A Streptococcus infection in children: protocol for a scoping review of the literature with a focus on predictors of invasive infection
Source: F1000Res. 2023 Feb 24;12:209. [Version 1] doi: 10.12688/f1000research.130989.1 (PMC11109578; doi:10.12688/f1000research.130989.1)
Supplement: Peer review history

# Reviewer report: Reviewer response for version 1

**DOI:** 10.5256/f1000research.143791.r279813

**Published:** 2024-05-21

**Authors:** Navin P Boeddha

**Affiliations:** 1 Department of Paediatrics, Division of Paediatric Infectious Diseases & Immunology, Erasmus MC-Sophia Children's Hospital, University Medical Center Rotterdam, Rotterdam, The Netherlands

This paper by Mariani and colleagues is a study protocol to review early signs and clinical features of iGAS in children. This is an important paper because early recognition and early treatment of invasive bacterial infections improve outcome. Apart from early recognition, the review will be much broader, e.g. epidemiology, therapy, and outcome.

 Overall the study protocol is presented clearly. A few minor comments/queries:

 1. Introduction, second paragraph: “It’s likely that this increase in incidence of iGAS is related to the high circulation of respiratory viruses, especially Respiratory Syncytial Virus (RSV) and seasonal influenza, since the coinfection of viruses can lead more easily to invasive disease.” Please provide a reference for this statement. I would suggest to weaken this statement as multiple factors may play a role, e.g. pathogenic emm types (Brouwer S, et al., 2023 [Ref1])

 2. Inclusion criteria: why do the authors do not include osteoarticular infections? (Trobisch A, et al., 2022 [Ref 2])

 3. Search strategy: is there a librarian involved?

 4. Data extraction: consider inclusion of the following variables: Immunization status (Varicella vaccination?), previous surgery (Duvekot A, et al., 2019 [Ref 3].), lab predictors  (CRP, PCT, leukocytes, neutrophils).

Is the study design appropriate for the research question?

Yes

Is the rationale for, and objectives of, the study clearly described?

Yes

Are sufficient details of the methods provided to allow replication by others?

Yes

Are the datasets clearly presented in a useable and accessible format?

Yes

Reviewer Expertise:

Pediatric Infectious Diseases and Immunology

I confirm that I have read this submission and believe that I have an appropriate level of expertise to confirm that it is of an acceptable scientific standard.

## References

[1] : Pathogenesis, epidemiology and control of Group A Streptococcus infection. *Nat Rev Microbiol* .2023;21(7) : 10.1038/s41579-023-00865-7 431-447 10.1038/s41579-023-00865-7 36894668 PMC9998027

[2] : Osteoarticular Infections in Pediatric Hospitals in Europe: A Prospective Cohort Study From the EUCLIDS Consortium. *Front Pediatr* .2022;10: 10.3389/fped.2022.744182 744182 10.3389/fped.2022.744182 35601438 PMC9114665

[3] : Subcutaneous and Mediastinal Emphysema Followed by Group A Beta-Hemolytic Streptococci Mediastinitis. A Complicated Course after Adenotonsillectomy: Case Report. *Diagnostics* .2019;9(1) : 10.3390/diagnostics9010011 10.3390/diagnostics9010011 30650516 PMC6468507

# Reviewer report: Reviewer response for version 1

**DOI:** 10.5256/f1000research.143791.r164677

**Published:** 2023-03-16

**Authors:** Damian Roland

**Affiliations:** 1 Paediatric Emergency Medicine Leicester Academic (PEMLA) Group, University Hospitals of Leicester NHS Trust, Leicester, UK; 2 SAPPHIRE Group, Health Sciences, University of Leicester, Leicester, UK

This is an important and timely piece of work. 

**Introduction: **

- Why was the increase in 2022 described as 'unexpectedly" when an increase was potentially inevitable after a reduction following lockdown?
- "It's likely this increase is related to high circulation of respiratory viruses" > This is obviously a possibility but why is this more likely than just the fact herd immunity and transmission waned during the pandemic?

**Concept:**

- " *The main concept of this review will be the iGAS in all its aspects*"
  > The focus of this review will be the impact iGAS in children and adolescents. 

**Search Strategy:**

- Why are only PubMed and SCOPUS being searched? Is there a reason for excluded COCHRANE and CINAHL? 
- Has a clinical librarian been involved? 

Is the study design appropriate for the research question?

Yes

Is the rationale for, and objectives of, the study clearly described?

Yes

Are sufficient details of the methods provided to allow replication by others?

Yes

Are the datasets clearly presented in a useable and accessible format?

Yes

Reviewer Expertise:

Paediatric Emergency Medicine Health Services Research

I confirm that I have read this submission and believe that I have an appropriate level of expertise to confirm that it is of an acceptable scientific standard.
